# Post-treatment with Posiphen Reduces Endoplasmic Reticulum Stress and Neurodegeneration in Stroke Brain

**DOI:** 10.1016/j.isci.2020.100866

**Published:** 2020-01-28

**Authors:** Seong-Jin Yu, Kuo-Jen Wu, Eunkyung Bae, Yu –Syuan Wang, Chia-Wen Chiang, Li-Wei Kuo, Brandon K. Harvey, Nigel H. Greig, Yun Wang

**Affiliations:** 1 Center for Neuropsychiatric Research, National Health Research Institutes, Zhunan, Taiwan; 2 Institute of Biomedical Engineering and Nanomedicine, National Health Research Institutes, Zhunan, Miaoli, Taiwan; 3 National Institute on Drug Abuse, NIH, Baltimore, MD, USA; 4 Translational Gerontology Branch, Intramural Research Program, National Institute of Aging, NIH, Baltimore, MD, USA

**Keywords:** Drugs, Molecular Biology, Clinical Neuroscience, Cell Biology

## Abstract

Acetylcholinesterase (AChE) inhibitors have protective and anti-inflammatory actions against brain injury, mediated by nicotinic α7 cholinergic receptor activation. The use of AChE inhibitors in patients is limited by systemic cholinergic side effects. Posiphen, a stereoisomer of the AChE inhibitor Phenserine, lacks AChE inhibitor activity. The purpose of this study is to determine the protective effect of Posiphen in cellular and animal models of stroke. Both Posiphen and Phenserine reduced glutamate-mediated neuronal loss in co-cultures of primary cortical cells and microglia. Phenserine-, but not Posiphen-, mediated neuroprotection was diminished by the nicotinic α7 receptor antagonist methyllycaconitine. Posiphen antagonized NMDA-mediated Ca^++^ influx, thapsigargin-mediated neuronal loss and ER stress in cultured cells. Early post-treatment with Posiphen reduced ER stress signals, IBA1 immunoreactivity, TUNEL and infarction in the ischemic cortex, as well as neurological deficits in stroke rats. These findings indicate that Posiphen is neuroprotective against stroke through regulating Ca^++^i and ER stress.

## Highlights

• Posiphen induces protection in cell culture through noncholinergic mechanism
• Posiphen attenuates glutamate-mediated Ca^++^i and ER stress in neuronal culture
• Posiphen mitigates ER stress in stroke brain
• Posiphen reduces neurodegeneration in stroke rats

## Graphical Abstract

**Figure:**
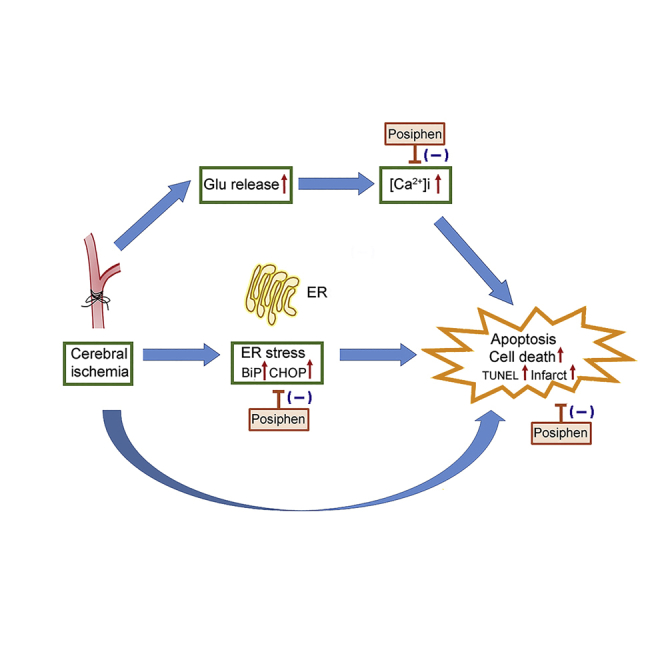

## Introduction

Stroke is a major brain disease worldwide, being the second leading global cause of death in the past decade (2000–2012; www.who.int/mediacentre/factsheets/fs310/en/index.html). Tissue plasminogen activator (tPA) is the only US FDA-approved pharmacological therapy for acute ischemic stroke. tPA dissolves occluding blood clots at a very early stage of stroke, but its effectiveness is limited by a narrow therapeutic time window of 3 h. Less than 3% of patients with stroke receive tPA, because they do not arrive at a hospital early enough for treatment (Barber et al., 2001, Reed et al., 2001). It is thus important to develop new therapies for stroke.

Cholinergic mechanisms are involved in various models of neurodegeneration including stroke (Pavlov and Tracey, 2005, Vijayaraghavan et al., 2013). Acetylcholine (ACh) ameliorated glutamate or N-methyl-D-aspartate (NMDA)-mediated toxicity in neuronal cells (Wehrwein et al., 2004, Dajas-Bailador et al., 2000) and mitigated cell death and inflammation via the cholinergic anti-inflammatory pathway (Vijayaraghavan et al., 2013, Pavlov and Tracey, 2005). The hydrolysis of ACh is enhanced after stroke (Ben et al., 2010), which results in reduced ACh levels (Haba et al., 1991, Tanaka et al., 1994) and a rise in the ACh metabolite choline in brain (Scremin and Jenden, 1989). Pretreatment with selective acetylcholinesterase (AChE) inhibitor Phenserine (Greig et al., 2005) reduced activated caspase 3, terminal deoxynucleotidyl transferase dUTP nick end labeling (TUNEL), and volume of infarction in the rat focal middle cerebral artery occlusion (MCAo) model (Chang et al., 2017). However, the efficacy of AChE inhibitor treatment depends on cholinergic integrity as seen in patients with early Alzheimer's disease (Richter et al., 2018) and the use of AChE inhibitors can be limited by the systemic cholinergic adverse effects (Colombres et al., 2004, Doody, 2003, Snape et al., 1999).

Posiphen (Pos), also known as (+)-Phenserine, a stereoisomer of Phenserine that cannot generate either Phenserine or its metabolites, has also been reported to increase cell survival (Lilja et al., 2013), protect against neurodegeneration in animal models of Alzheimer's disease, and reduce neuroinflammation (Yu et al., 2013, Maccecchini et al., 2012, Teich et al., 2018). Unlike Phenserine, Posiphen is devoid of cholinesterase (ChE) inhibition activity or cholinergic side effects (Klein, 2007). Posiphen also does not bind to muscarinic or nicotinic cholinergic receptors (Lahiri et al., 2007, Lecca et al., 2019). As such, it appears to be distinct from various other Alzheimer's disease drugs and potential treatments, such as Phenserine, that reduce degeneration through multiple mechanisms that include the cholinergic anti-inflammatory pathway (Pavlov et al., 2009, Han et al., 2017). The potential protective action against stroke has not been reported for Posiphen.

The endoplasmic reticulum (ER) is essential for the maintenance of intracellular protein function. The ER controls the folding of newly synthesized proteins into their mature conformation and the transport of matured/secreted proteins to other compartments within the cells. The ER also serves as the primary intracellular reservoir for calcium, and reducing ER Ca^++^ causes ER stress. We previously reported a method to monitor ER calcium homeostasis through the use of secreted ER calcium—monitoring proteins (SERCaMPs) in SH-SY5Y cells (Henderson et al., 2014). This construct also includes the reporter Gaussia luciferase (GLuc) thus forming GLuc-SERCaMP (Chen et al., 2019). In cells expressing GLuc-SERCaMP, the ER stressor thapsigargin (Tg) or methamphetamine induces secretion of GLuc (Chen et al., 2019). Systemic application of Tg increased GLuc release into plasma in rats receiving an intrahepatic injection of AAV-GLuc-SERCaMP (Wires et al., 2017). These data suggest that GLuc-SERCaMP is a useful pharmacological tool for monitoring ER stress in cell culture or *in vivo*.

Several genetically encoded Ca^++^ indicators (GECIs) have been developed to monitor intracellular Ca^++^ (Ca^++^*i*) in neurons and non-neuronal cells (Mao et al., 2008, Shigetomi et al., 2010, Tian et al., 2009). gCaMP is a commonly used GECI probe, which consists of a single circularly permuted green fluorescent protein (GFP), calmodulin (CaM), and an M13 fragment from myosin light-chain kinase. Binding of Ca^++^ to CaM induces conformational changes of gCaMP and results in increased fluorescence intensity in cells (Nakai et al., 2001). We previously delivered gCaMP5 to cultured neuronal cells using an adeno-associated viral (AAV) vector (Akerboom et al., 2012). We demonstrated that methamphetamine enhanced Ca^++^*i* in real time in neurons, which was antagonized by MK801, Mg^++^, or the ryanodine receptor (RyR) inhibitor dantrolene.

In the present study, we examined the neuroprotective actions of Phenserine and its non-cholinergic stereoisomer Posiphen in cellular and rodent models of stroke. Both Posiphen and Phenserine reduced glutamate-mediated neurodegeneration. Posiphen selectively mitigated thapsigargin (Tg)-mediated neuronal death and the release of ER stress markers in cells over-expressing GLuc-SERCaMP as well as the NMDA–mediated increase in Ca^++^*i*. Early post-ischemia treatment with Posiphen reduced the calcium-binding adaptor molecule 1 (IBA1) immunoreactivity, TUNEL activity, brain infarction, and the expression of ER stress markers in the lesioned cortex in stroke rats. These data suggest that Posiphen reduces ischemic neuronal injury through regulating Ca^++^_*i*_ and ER stress.

## Results

### Posiphen and Phenserine Elicit Neuroprotection in Primary Cortical Cells and Microglia Co-cultures

**Figure 1:**
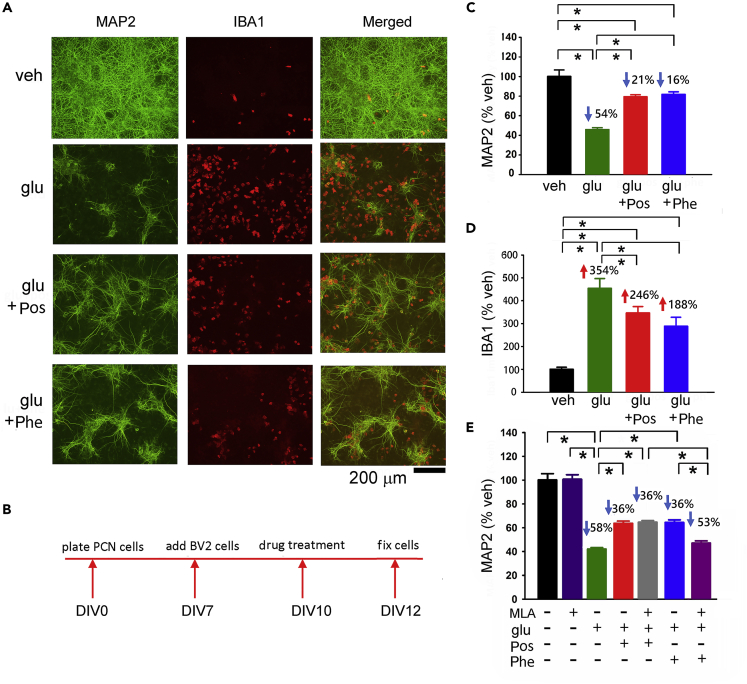
Both Posiphen and Phenserine Reduce Glutamate-Induced Neuronal Degeneration in Primary Cortical Neuronal and BV2 Microglial Co-culture (A) Representative photomicrographs demonstrate that treatment with glutamate reduced MAP2 while increasing IBA1 immunoreactivity in microglia (red) and neurons (MAP2 staining in green color) co-culture. Scale bar, 200 μm. (B–D) (B) Timeline of the experiment. Immunoreactivity of MAP2 (C) and IBA1(D) were normalized to the mean of the control samples. Posiphen and Phenserine (15 μM) significantly antagonized glutamate (100 μmol/L)-mediated changes in MAP2 (*p < 0.001, one-way ANOVA + NK test) and IBA1 immunoreactivity (*p < 0.001, one-way ANOVA on Rank; *p < 0.05, post hoc NK test). (E) Co-treatment with the nicotinic α7 receptor antagonist methyllycaconitine (MLA) significantly antagonized Phenserine- (Glu + Phe versus Glu + Phe + MLA, *p = 0.004, one-way ANOVA + NK test), but not Posiphen- (Glu + Pos versus Glu + Pos + MLA, p = 0.980), mediated protection. The numbers above each response in (C)–(E) represent the percentage decrease or increase, relative to the veh control. (C) and (D): n = 7 in each group; (E): n = 5–8 per group. “n” represents the number of replicates. Data are represented as mean ± SEM.

We first examined Posiphen- and Phenserine-mediated neural protection in a mixed primary cortical cell and BV2 microglial co-culture. A high dose (100 μM) of glutamate (Glu) was used to generate neurodegeneration and inflammation (Wu et al., 2017, Choi et al., 1987) and to simulate overflow of elevated glutamate during cerebral ischemia (Shen et al., 2005). Glutamate significantly reduced the neuronal marker Microtubule-Associated Protein 2 (MAP2; Figures 1A and 1C, p < 0.001), associated with an elevation in ionized calcium-binding adapter molecule 1 (IBA1) immunoreactivity (Figures 1A and 1D, p < 0.001). Both responses were significantly mitigated by 15 μM Phenserine (MAP2, p < 0.001; IBA1: p = 0.005) or by Posiphen (MAP2, p < 0.001; IBA1: p = 0.05) as seen in Figures 1C and 1D (timeline of experiment: Figure 1B). Co-treatment with the nicotinic α7 receptor antagonist methyllycaconitine (MLA, at 500 nM) significantly attenuated Phenserine- (Figure 1E, Glu + Phe versus Glu + Phe + MLA, p = 0.004), but not Posiphen- (Glu + Pos versus Glu + Pos + MLA, p = 0.980; Glu versus Glu + Pos + MLA, p < 0.001), mediated protection, suggesting a non-cholinergic protective action of Posiphen and a cholinergic component for the neuroprotective actions of Phenserine.

### Posiphen Reduces Glutamate Neurotoxicity in Primary Cortical Neuronal Cultures

**Figure 2:**
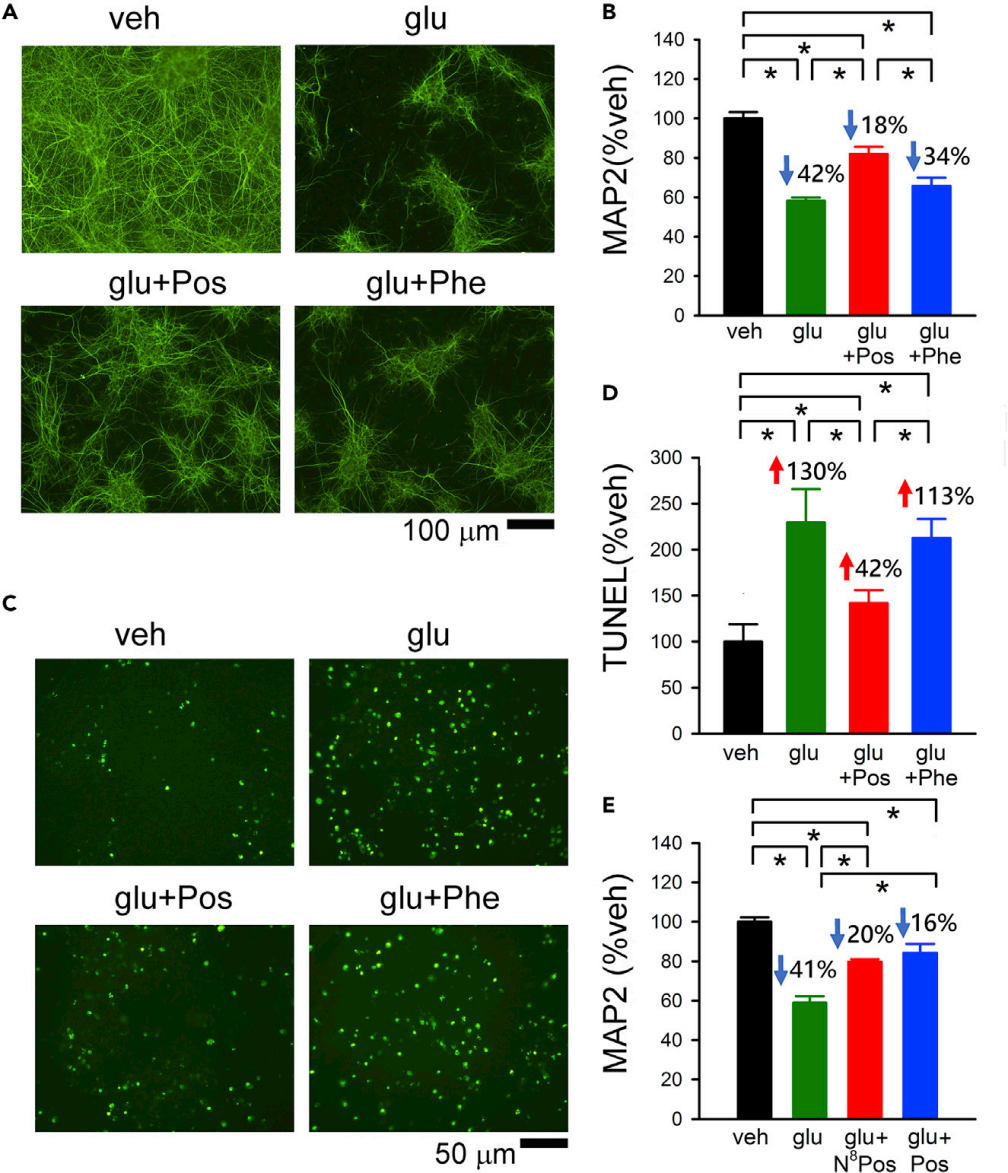
Posiphen, but Not Phenserine, Antagonized Glutamate-Mediated Neuronal Loss and TUNEL Labeling in Primary Cortical Neuronal Culture (A and C) Representative photomicrographs demonstrate that Posiphen antagonized glutamate-mediated (A) loss of MAP2 immunoreactivity and (C) an increase in TUNEL labeling. (B) Posiphen, but not Phenserine, significantly antagonized glutamate- (100 μM) mediated neuronal loss (*p < 0.001, Glu versus Glu + Posiphen; p = 0.107, Glu versus Glu + Phenserine, F_3,24_ = 33.168, one-way ANOVA + NK test, n = 7 in each group). (D) Posiphen significantly antagonized glutamate-mediated increased TUNEL activity (*p < 0.001, Glu versus Glu + Posiphen; p = 0.212, Glu versus Glu + Phenserine, F_3,26_ = 47.867, one-way ANOVA + NK test, n = 6–9 in each group). (E) (+)-N8-NorPosiphen (15 μmol/L), a metabolite of Posiphen with no anti-AChE activity, also antagonized glutamate-mediated loss of MAP2 immunoreactivity (*p < 0.001, F_3,20_ = 29.986, n = 6 in each group). “n” represents the number of replicates. Data are represented as mean ± SEM. Scale bar: 100 μm in (A) and 50 μm in (C).

Since inflammation mainly occurs in the neuron + astrocyte/microglia co-culture, we next examined the protective effect of Posiphen and Phenserine 15 μM in primary cortical neuronal culture in the absence of microglia. Posiphen selectively antagonized glutamate-mediated neuronal loss (Figures 2A and 2B, Glu versus Glu + Posiphen, p < 0.001). A mild, but not significant, improvement in MAP2 immunoreactivity was found after Phenserine treatment (Glu versus Glu + Phe, p = 0.107). A significant difference was also found between Glu + Pos and Glu + Phe (p = 0.001, Figure 2B). Glutamate-mediated apoptosis was examined by TUNEL labeling (Figure 2C). Posiphen, but not Phenserine, significantly reduced glutamate-enhanced TUNEL activity in the neuronal culture (Figure 2D, p < 0.001).

(+)-N^8^-NorPosiphen is a major metabolite of Posiphen found in brain and plasma after systemic administration of Posiphen (Teich et al., 2018). (+)-N^8^-NorPosiphen, similar to Posiphen, does not bind to cholinergic receptors and has no anti-AChE activity in peripheral erythrocytes (Yu et al., 2013). Treatment with (+)-N^8^-NorPosiphen (15 μM) significantly antagonized glutamate-mediated loss of MAP2 immunoreactivity in primary neuronal culture (p < 0.001, F_3,20_ = 29.986, one-way ANOVA + Newman-Keuls [NK] test, Figure 2E), suggesting (+)-N8-NorPosiphen is an active metabolite of Posiphen.

### Posiphen Alters Intracellular Ca^++^

**Figure 3:**
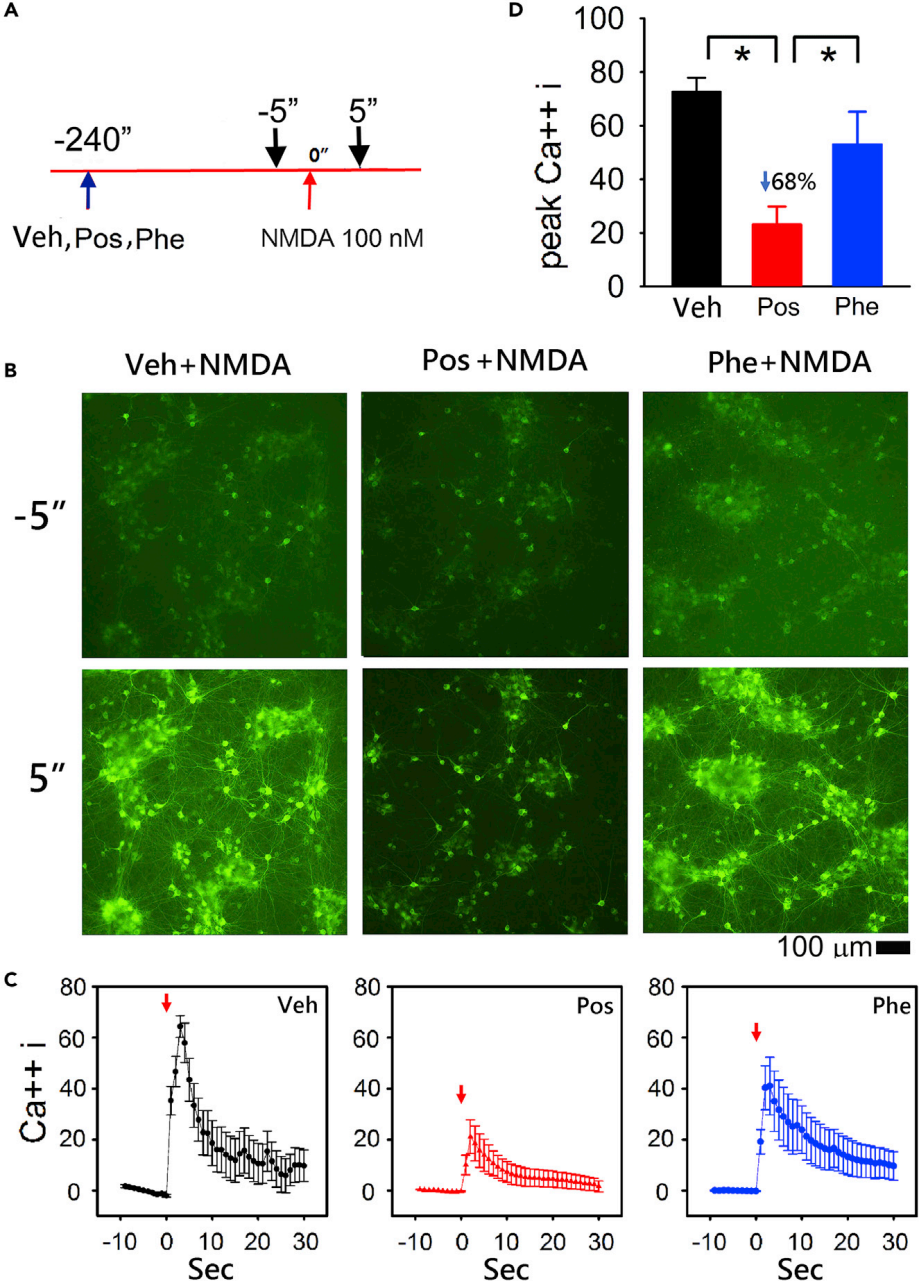
Posiphen Reduced NMDA-induced Ca^++^_*I*_ in Primary Cortical Neuronal Culture Overexpressing gCaMP5 The Calcium probe gCaMP5 was overexpressed in the primary cortical neurons through AAV infection. (A) Timeline of drug treatment. Posiphen or Phenserine (15 μM) was given (blue arrow) 4 min before administration of NMDA (100 nM, red arrow, time 0). Typical real-time intracellular Ca^++^ concentration (Ca^++^*i*) fluorescence images were taken at 5 s before and 5 s (black arrows) after NMDA administration. (B) Administration of NMDA triggered a rapid increase in intracellular Ca^++^ as indicated by increasing intracellular green fluorescence at 5 s after NMDA administration (left lower panel), compared with the image taken 5 s before drug treatment (left upper panel). Pretreatment with Posiphen (middle panels), but not Phenserine (right panels), suppressed NMDA-mediated increased intracellular Ca^++^ signals. Scale bar, 100 μm. (C) The intensity of fluorescence (% maximal response) was analyzed in each well every second. Administration of NMDA (red arrow at time 0) significantly increased Ca^++^*i*, as compared with the response before NMDA administration (n = 13). Pretreatment with Posiphen (n = 8) significantly antagonized NMDA-mediated increases in Ca^++^*i* (Pos + NMDA versus NMDA: p < 0.001, two-way ANOVA + NK test). Significant reductions in Ca^++^*i* after Posiphen treatment occurred between 1 and 17 s after administration of NMDA (*p = 0.001–0.047, two-way ANOVA + NK test). Treatment with Phenserine (n = 8) did not alter NMDA-mediated Ca^++^_*I*_ (Phe + NMDA versus NMDA: p = 0.619). (D) The peak Ca^++^*i* was significantly suppressed after Pos treatment (*p < 0.001). “n” represents the number of replicates. Data are represented as mean ± SEM.

We previously measured glutamate-mediated increase in intracellular Ca^++^ signals in real time in primary cortical neurons overexpressing an intracellular Ca^++^ probe, gCaMP5, by AAV serotype 1 (Yu et al., 2016). Utilizing this approach we examined NMDA-mediated changes in Ca^++^*i* in primary cortical cultures. Cultured cells were infected with AAV1-gCaMP5 on DIV5. Intracellular Ca^++^, as indicated by a change in intracellular green fluorescence, was monitored on DIV 10. Representative interactions between Posiphen, Phenserine, and NMDA are shown in the captured live images (Figure 3B). NMDA at 100 nM induced a rapid increase in intracellular Ca^++^ as indicated by increasing intracellular green fluorescence (Figure 3B, left lower panel). Peak fluorescence occurred within 3–6 s after treatment. Posiphen or Phenserine (15 μM) was given to cells 4 min before the application of NMDA (timeline, Figure 3A). Pretreatment with Posiphen (Figure 3B, middle panels), but not Phenserine (Figure 3B, right panels), suppressed NMDA-mediated intracellular Ca^++^ signals. The temporal changes in intensity of intracellular gCaMP5 fluorescence was analyzed in each well every second from −5 to 30 s (Figure 3C). NMDA significantly increased Ca^++^ fluorescence intensity (p < 0.01, n = 13). Pretreatment with Posiphen (n = 8), but not Phenserine (n = 8), significantly attenuated NMDA-induced increases in Ca^++^*i* (Pos + NMDA versus NMDA: p < 0.001; Pos + NMDA versus Phe + NMDA: p < 0.001; Phe + NMDA versus NMDA: p = 0.619, Figure 3C). The reductions in Ca^++^*i* after Posiphen treatment were statistically significant between 1 and 6 s after administration of NMDA (p = 0.001–0.036, two-way ANOVA + NK test, Figure 3C). The peak Ca^++^*i* was significantly suppressed after Pos treatment (p < 0.001, Figure 3D).

### Posiphen Reduces ER Stress in Cultured SH-SY5Y and Primary Cortical Cells

**Figure 4:**
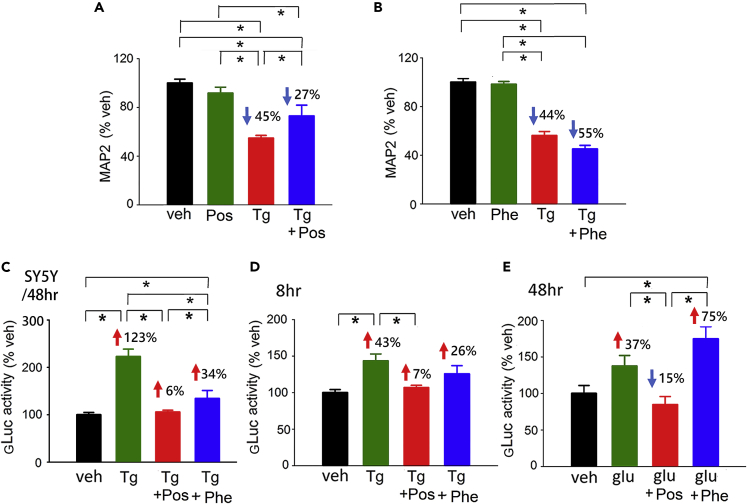
Posiphen Reduces ER Stress in Cell Culture (A and B) MAP2-ir in the primary cortical neuronal culture was quantified after treatment with Tg (500 nmol/L) with or without (A) Posiphen and (B) Phenserine (15 μmol/L). Tg significantly reduced MAP2 immunoreactivity. This response was reduced by (A) Posiphen (*p = 0.026, one-way ANOVA + NK test), but not by (B) Phenserine (*q = 2.673, one-way ANOVA on Rank + NK test). (C–E) (C) The ER stress sensor GLuc-SERCaMP was overexpressed in the SH-SY5Y cells. Tg (500 nmol/L) significantly increased GLuc-SERCaMP release to the culture media at 48 h after drug treatment (*p = 0.002, Tg versus veh, one-way ANOVA + NK test). Co-treatment with Posiphen (15 μmol/L) significantly reduced Tg-mediated GLuc release (*p < 0.001). Similarly, GLuc-SERCaMP was overexpressed in primary cortical neurons by AAV infection (D and E). (D) Tg (500 nM) or (E) glutamate (100 μM) significantly increased the release of GLuc-SERCaMP into the media at 8 (*p = 0.002) and 48 h (*p < 0.001) post-treatment, respectively. Posiphen significantly reduced Tg ([D]: Tg + Pos versus Tg, *p = 0.001, one-way ANOVA + NK test) and Glu-mediated secretion of GLuc ([E]: Glu + Pos versus Glu, *p = 0.016). Phenserine did not reduce (D) Tg (*p = 0.153) or (E) Glu-mediated gLuc activity. ([A], [B], [C]: n = 6–8; [D], [E], n = 4–8 in each group). “n” represents the number of replicates. Data are represented as mean ± SEM.

As disturbance of intracellular and ER Ca^++^ homeostasis can lead to ER stress (Krebs et al., 2015, Williams et al., 2013), we next examined the interaction of Posiphen with the ER stressor Tg in primary cortical cell cultures. Treatment with Tg (500 nM) significantly reduced MAP2 immunoreactivity (Figure 4A and 4B, p < 0.001). This response was partially antagonized by Posiphen (Figure 4A, p = 0.026), but not by Phenserine (each 15 μM) (Figure 4B, q = 2.673, one-way ANOVA on rank + NK test).

Tg-mediated ER stress was also examined in SH-SY5Y cells expressing GLuc-SERCaMP, a reporter for ER stress and proteostasis (Trychta et al., 2018) that is triggered by ER calcium depletion (Henderson et al., 2014). Treatment with Tg significantly increased luciferase (GLuc) activity in media collected 48 h after drug treatment (Figure 4C, p = 0.002, Tg versus veh). Co-treatment with Posiphen effectively blocked Tg-induced GLuc activity (p < 0.001). Phenserine also reduced the Tg-induced GLuc activity (Tg versus Phe + Tg, p = 0.001, Figure 4C) but was less effective than Posiphen (Pos + Tg versus Phe + Tg, p = 0.002, Figure 4C).

Tg-mediated GLuc-SERCaMP release was also examined in primary cortical cells expressing GLuc-SERCaMP on DIV13 (Figures 4D and 4E). Tg or glutamate significantly increased the secretion of GLuc at 8 or 48 h post-treatment, respectively (Figure 4D, p = 0.002; E: p < 0.001). Both Tg and glutamate responses were antagonized by Posiphen (Figure 4D, Tg + Pos versus Tg, p = 0.001; Figure 4E, Glu + Pos versus. Glu, p = 0.016). Phenserine did not significantly alter Tg- (p = 0.153, Figure 4D) or glutamate-mediated secretion of GLuc (Figure 4E).

### Neuroprotective Action of Posiphen in Stroke Rats

**Figure 5:**
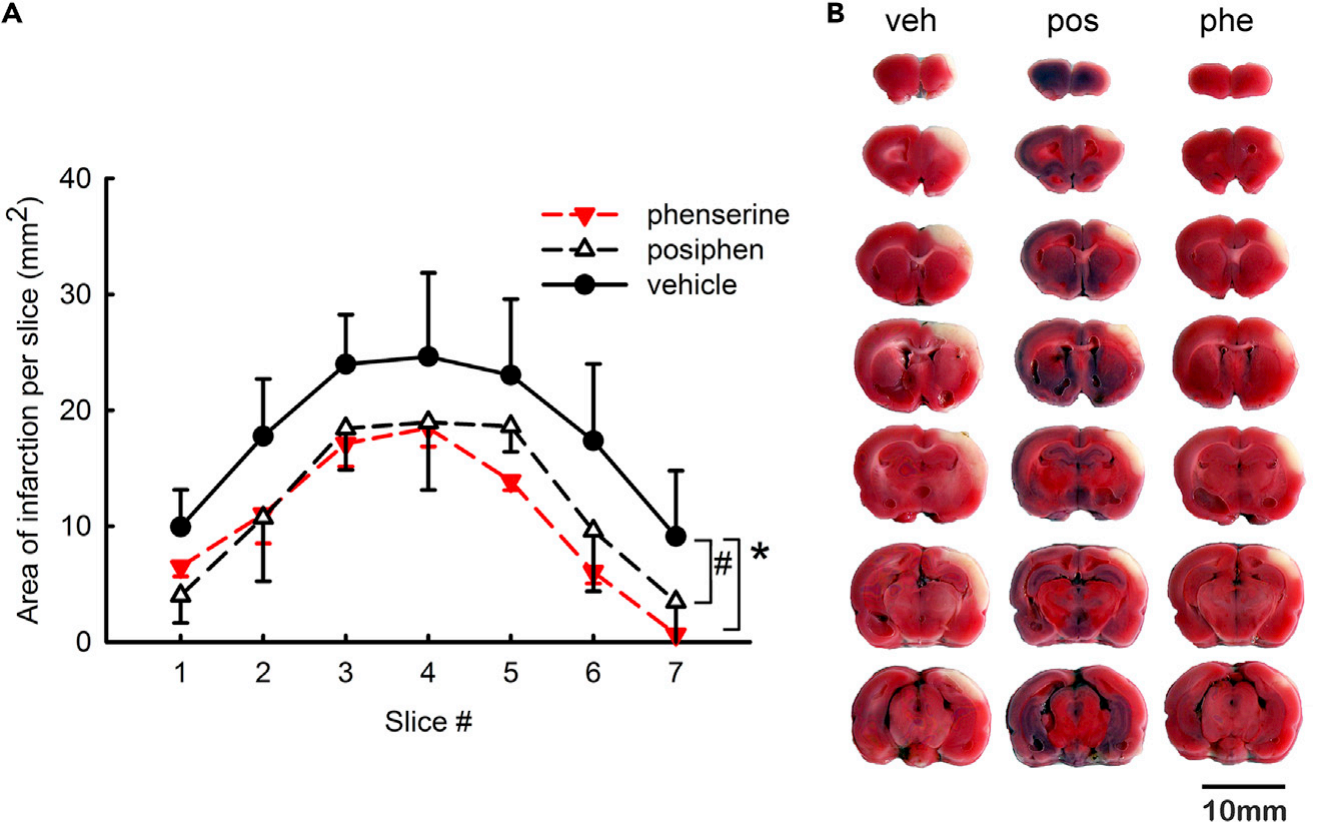
Post-treatment with Posiphen or Phenserine Reduced Brain Infarction in Stroke Rats Animals received a 60-min middle cerebral artery occlusion (MCAo) on day 0. (A) Phenserine (1 mg/kg/day, i.p., n = 4), Posiphen (25 mg/kg/day, i.p., n = 3), or vehicle (n = 3) were given to the stroke rats daily for 4 days after the MCAo. Brains were sliced into 2.0-mm-thick sections on day 5. The area of infarction in brain slices was analyzed after TTC staining. Posiphen or Phenserine significantly reduced brain infarction at 5 days after the MCAo (*, ^#^ p < 0.01, two-way ANOVA + NK test). (B) Typical TTC images from rats receiving vehicle (veh), Posiphen (Pos), or Phenserine (Phe). Scale bar, 10 mm. “n” represents the number of animals used. Data are represented as mean ± SEM.

**Figure 6:**
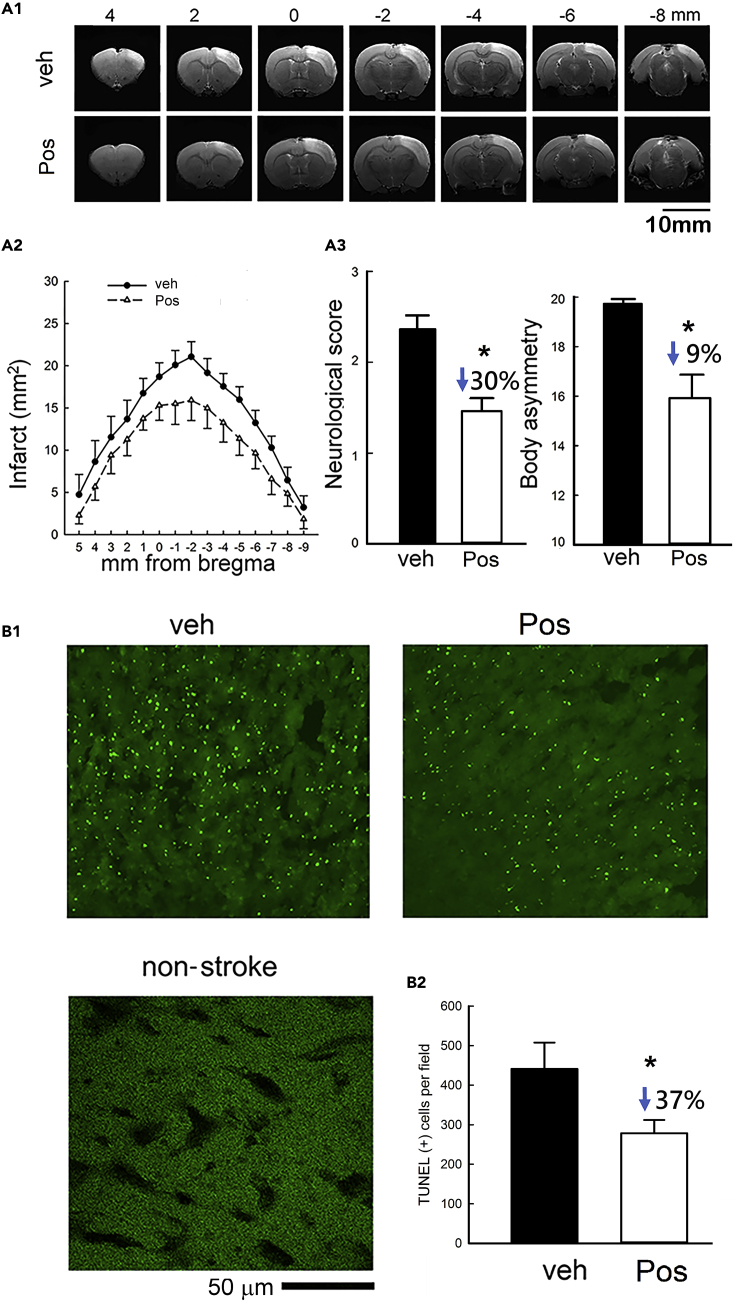
Post-treatment with Posiphen Reduced Neurodegeneration in Stroke Rats Adult rats received a 60-min MCAo. Posiphen (25mg/kg, i.p.) or veh was given 30 min after the MCAo and the following day (days 0 and 1). (A1) Representative T2WI from two stroke rats receiving with vehicle (upper) or Posiphen (lower). Scale bar, 10 mm. (A2) The area of infarction was averaged in each millimeter in stroke rats receiving vehicle (n = 8) or Posiphen (n = 8). Posiphen treatment significantly reduced brain infarct size (*p < 0.001, two-way ANOVA + NK test). (A3) Animals received neurological tests 2 days after MCAo. Post-treatment of Posiphen significantly reduced Bederson's neurological score (*p = 0.001; Mann-Whitney rank-sum test) and body asymmetry (*p = 0.0014, t test). (B1) Brain tissue was collected on day 3 for TUNEL labeling. Scale bar, 50 μm. (B2) Posiphen treatment reduced TUNEL (+) cell density in the peri-lesioned area (*p = 0.048, veh n = 3; Pos, n = 5, t test). “n” represents the number of animals used. Data are represented as mean ± SEM.

Pretreatment with Phenserine has been reported to reduce brain infarction volume (Chang et al., 2017). Phenserine and Posiphen were first examined here for stroke protection activity using standard triphenyl tetrazolium chloride (TTC) staining. Animals received a 60-min MCAo on day 0. Phenserine (1 mg/kg/day, i.p.) or Posiphen (25 mg/kg/day, i.p.) was administered daily as a post-treatment for 4 days (from day 1 to day 4) after MCAo. Both Posiphen and Phenserine significantly reduced brain infarction on day 5 (p < 0.01, n = 10, Figure 5). Phenserine is dose limited at 25 mg/kg by its cholinergic actions, whereas Posiphen is not and can hence be administered at this and higher doses (Lahiri et al., 2007). All animals receiving Posiphen (25 mg/kg) tolerated the dose well prior to TTC staining. The protective effect of Posiphen was further examined by MRI T2WI in 16 rats (Figure 6A). Posiphen (25 mg/kg/day, i.p.) or vehicle was administered twice, at 1 h and 1 day after MCAo. An increase in T2WI signal intensity was found in the cortex on the lesioned side 2 days after MCAo. Typical T2WIs from two rats receiving Posiphen or vehicle are illustrated in Figure 6A1. Post-treatment with Posiphen significantly reduced brain infarct size (p < 0.001, Figure 6A2). Two neurological tests were used to examine behavioral improvement in 24 stroke rats. In an elevated body swing test, Posiphen treatment significantly reduced body asymmetry in 20 trials (*p = 0.0014, vehicle, n = 11, Posiphen, n = 13, t test, Figure 6A3). Posiphen also significantly attenuated neurological deficits, examined by Bederson's neurological test, in stroke rats (p = 0.001; vehicle, n = 11, Posiphen, n = 13, Mann-Whitney rank-sum test, Figure 6A3).

Animals were euthanized and perfused on day 3 for TUNEL and IBA1 immunohistochemical assays. TUNEL activity was quantified and averaged in three consecutive brain sections at the level of the anterior commissure for each animal (Figure 6B1). Posiphen significantly reduced TUNEL-positive cell density (Figure 6B2, p = 0.048, veh n = 3; Pos, n = 5).

**Figure 7:**
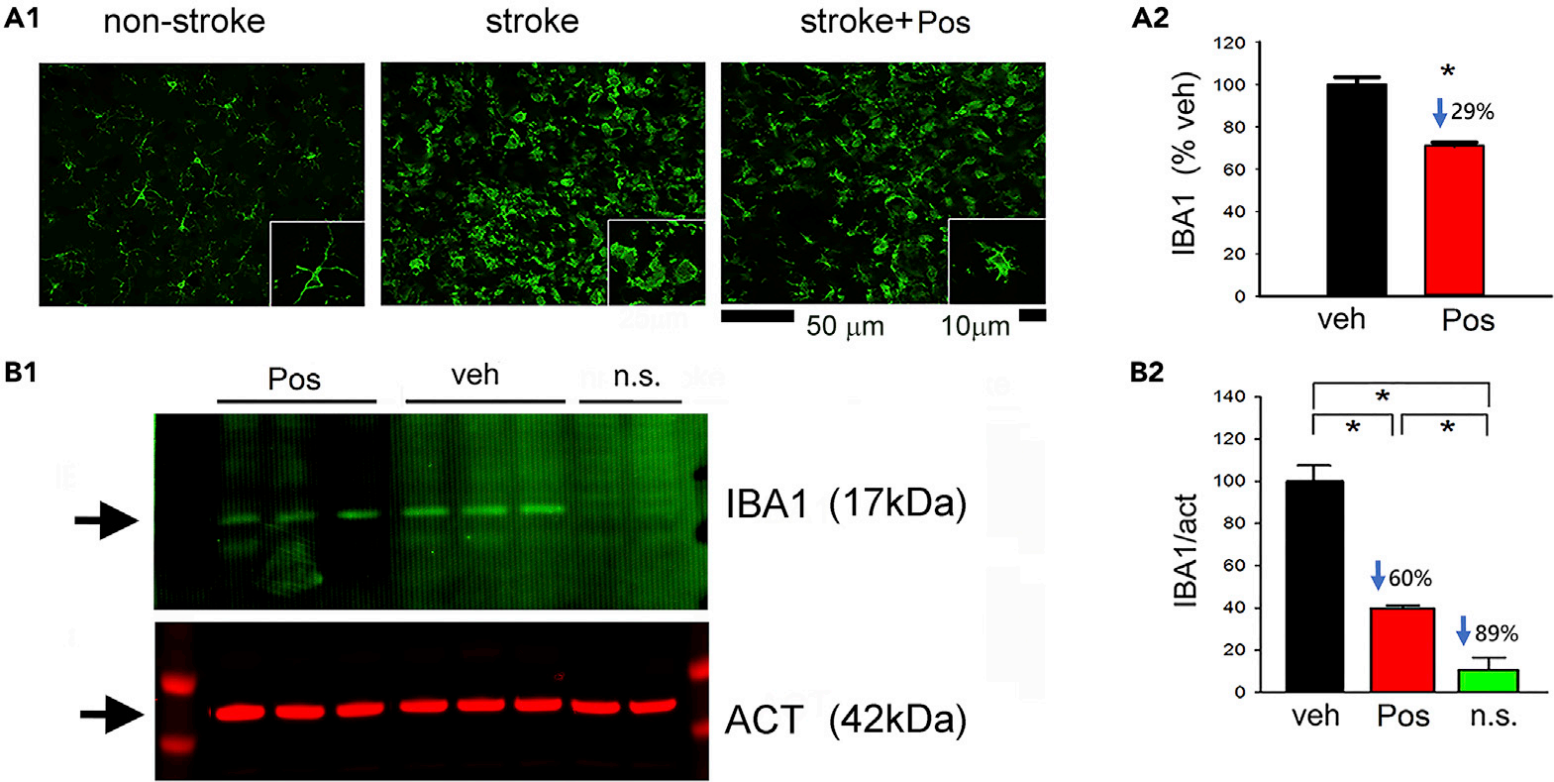
Post-treatment with Posiphen Reduced Microglia Activation in the Peri-Lesioned Area (A1) Minimal IBA1-ir was present in a non-lesioned rat (left panel). Enhanced IBA1-ir in microglia (middle panel, scale bar, 50 μm) with ameboid morphology (inset, scale bar, 10 μm) was found in a stroke animal receiving vehicle. Posiphen (25mg/kg, i.p.) treatment reduced IBA1 immunoreactivity (right panel). High-magnification image indicated that resting microglia exhibited ramified morphology in the non-lesioned side cortex (inset). (A2) IBA1-ir in all stroke brains (n = 7) was quantified. Posiphen treatment significantly reduced IBA1-ir (*p < 0.01, t test). (B1) Western blot analysis indicated that Posiphen reduced IBA1 immunoreactivity in the ischemic cortex. (B2) The effect of Posiphen on IBA1 expression was statistically significant, as determined by western blotting (*p < 0.05, one-way ANOVA, n = 8). n.s. = no stroke. “n” represents the number of animals used. Data are represented as mean ± SEM.

Enhanced IBA1 immunoreactivity was found in the peri-lesioned cortical area in stroke animals receiving vehicle (Figure 7A1). Microglia exhibited a ramified morphology in the non-lesioned cortex (Figure 7A1, left panel, inset), whereas rounded or ameboid microglial cells were found in the lesioned cortex in stroke animals receiving vehicle (Figure 7A1, middle panel, inset). Posiphen reduced IBA1 immunoreactivity (Figure 7A1) as well as morphological evidence of activation of microglia in the peri-lesioned area (Figure 7A1, right panel, inset). Partially ramified microglia were also found in the peri-lesioned area in animals receiving Posiphen (Figure 7A1, right panel, inset). The averaged IBA1 optical density in the peri-lesioned zone was significantly reduced by Posiphen (p < 0.01, Figure 7A2). The reduction of IBA1 activity by Posiphen was also confirmed by western blot analysis (Figure 7B1). Cortical brain tissue was collected from eight rats on day 2. Posiphen significantly reduced IBA1 protein expression in the lesioned cortex (Figure 7B2, p < 0.01).

### Detection of ER Stress in Stroke Brain

**Figure 8:**
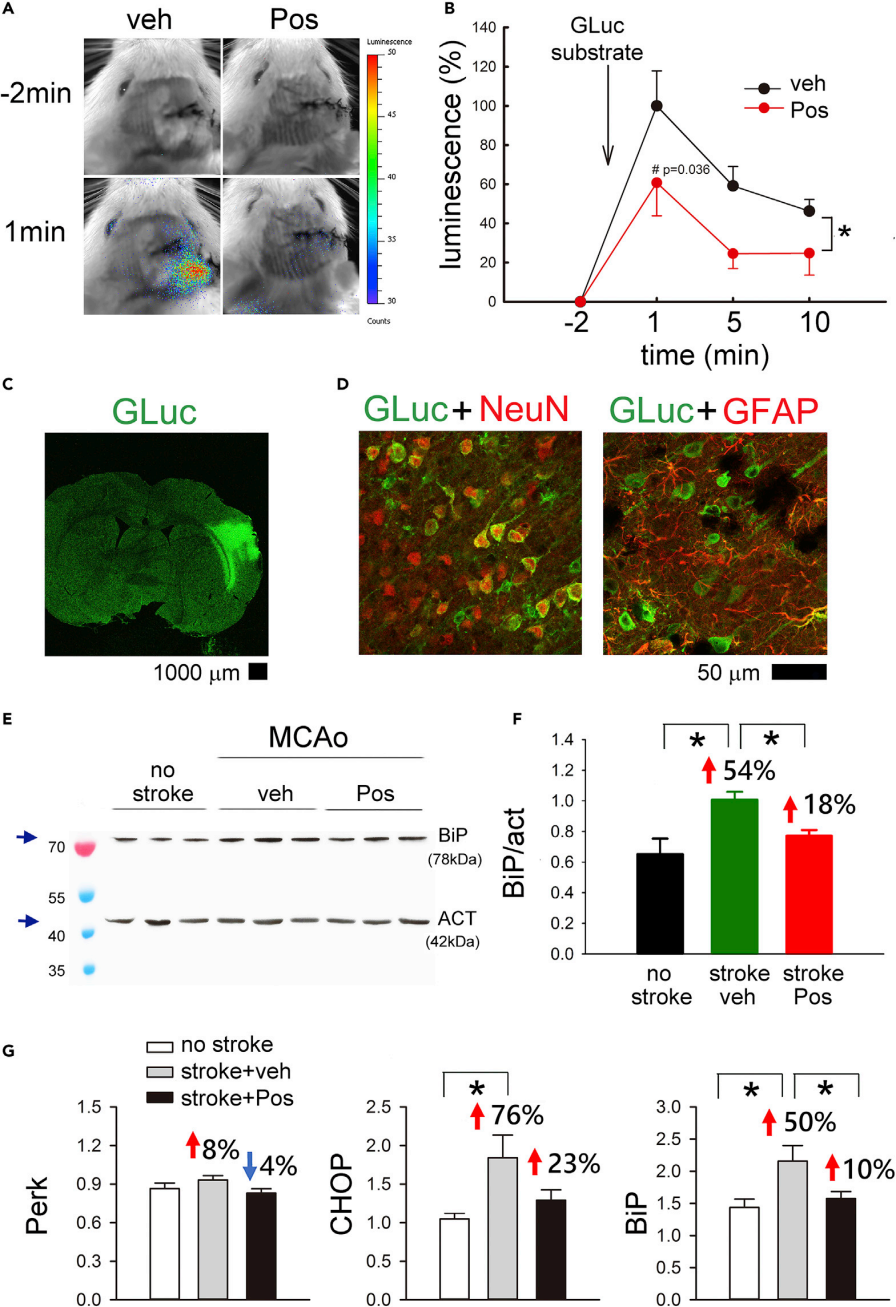
Posiphen Suppressed ER Stress in Stroke Brain (A) Posiphen (25mg/kg, i.p.) reduced release of ER stress marker GLuc-SERCaMP from the lesioned brain at 1 day after a 30-min MCAo. Adult rats received intracerebral administration of AAV-GLuc SERCaMP 2 weeks before the MCAo. GLuc activity was determined by IVES after administration of Coelenterazine (i.v. through the tail vein). Peak GLuc activity was found 1 min after injection of Coelenterazine in a stroke rat receiving veh (left panel, blue color) and was reduced by Posiphen (right panel). (B) *In vivo* GLuc activity was averaged from all animals (n = 10) studied. Treatment with Posiphen (red, n = 5) significantly attenuated GLuc activity in the stroke brain (*p = 0.007, two-way ANOVA + NK test; ^#^p = 0.036). (C) Cerebral tissues were collected after IVIS scanning for gLuc immunostaining. GLuc immunoreactivity was found in the cortex near the AAV injection sites. Scale bar, 1,000 μm. (D) GLuc-ir was found mainly in NeuN (+) cells. GLuc-ir was not co-expressed in GFAP cells. Scale bar, 50 μm. (E) Cerebral cortex was collected at 1 day after a 30-min MCAo and drug treatment for western blotting of BiP. Enhanced BiP immunoreactivity was found in stroke rats receiving vehicle, as compared with no stroke control. (F) Posiphen significantly reduced BiP expression (normalized to Act) in the lesioned side cortex (*p = 0.017, Posiphen, n = 6; veh, n = 6, non-stroke, n = 5). (G) Cortex was collected from 8 non-stroke rats and 16 stroke rats (Posiphen, n = 8; veh, n = 8) at 2 days after a 60-min MCAo for qRTPCR analysis. Expression of the target genes, PERK, CHOP, and BiP, was calculated relative to endogenous reference genes (average of Beta-actin and GAPDH). BiP and CHOP, but not Perk, were significantly upregulated in the ischemic side cortex (BiP: *p = 0.007, CHOP: *p = 0.006; one-way ANOVA + NK test). Post-treatment with Posiphen significantly reduced the expression of BiP (*p = 0.025). CHOP was marginally attenuated by Posiphen treatment (p = 0.056). “n” represents the number of animals used. Data are represented as mean ± SEM.

The ability of Posiphen to mitigate ER stress was next examined using GLuc-based SERCaMP. A total of 10 rats received intracerebral administration of AAV-GLuc SERCaMP 2 weeks before MCAo. Posiphen (n = 5) or vehicle (n = 5) was given i.p. 1 h and 1 day after a 30-min MCAo. Animals were anesthetized 60 min after the second dose of Posiphen for an *in vivo* imaging system (IVIS) scanning. The GLuc substrate Coelenterazine (100 μg/150μL) was administered intravenously through the tail vein. A minimal GLuc luminescence signal was detected before injection of Coelenterazine (Figure 8A). Enhanced GLuc activity was found 1 min after substrate administration, gradually declining over 10 min. Posiphen significantly attenuated GLuc activity (Figure 8B, p = 0.007). No GLuc activity was found in control animals without MCAo. Cerebral tissues were collected after IVIS scanning for gLuc immunostaining. GLuc immunoreactivity was found in the cortex near the AAV injection sites (Figure 8C). As seen in Figure 8D, Gluc was expressed mainly in neuronal cells. Cerebral cortical tissues were also collected from 12 rats at 1 day after a 30-min MCAo (vehicle, n = 6; Posiphen, n = 6) and 5 non-stroke rats for the ER stress marker BiP protein analysis (Figures 8E and 8F). Posiphen (n = 6) significantly reduced the expression of BiP in the cortex on the lesioned side (p = 0.017, Figures 8E and 8F).

Another set of animals (nonstroke, n = 8, stroke + veh, n = 8, stroke + Posiphen, n = 8) was used to examine ER stress markers by qRTPCR. Cortical tissue was collected 2 days after a 60-min MCAo. BiP and Chop, but not Perk, were significantly upregulated in the cortex on the ischemic side (Figure 8G, BiP, p = 0.007; Chop, p = 0.006). Post-treatment with Posiphen reduced expression of BiP (p = 0.025) and marginally attenuated Chop expression (p = 0.056). In the contralateral cortex, expression of Chop or BiP was not altered by MCAo or Posiphen treatment (data not shown). Perk, however, was significantly upregulated in the contralateral cortex after MCAo (data not shown).

## Discussion

We characterized the protective effect of Posiphen in neuronal cultures and experimental animals. Posiphen reduced the glutamate-mediated loss of MAP2-ir, the glutamate-induced increased TUNEL staining, and the glutamate-induced microglia activation in primary neuronal cultures. Parallel neuroprotective effects were seen in animals, since early post-stroke treatment with Posiphen significantly improved behavioral function, reduced brain infarction, and suppressed expression of inflammatory and ER stress markers in stroke rats. Thus, Posiphen is a potent non-cholinergic neuroprotective agent against ischemic stroke in animals.

CNS cholinergic activity is altered by stroke. For example, ACh levels in hippocampus are decreased following stroke in rats (Haba et al., 1991, Tanaka et al., 1994). The binding of the cholinergic ligands, [^3^H]quinuclidinyl benzilate or pirenzepine, to muscarinic receptors was found reduced up to 14 days after transient forebrain ischemia in the gerbil (Onodera et al., 1987, Hirata et al., 1992). Nicotinic ACh receptors (alpha 7R) were significantly down-regulated after hypoxia-ischemia injury in the neonatal mouse brain (Hua et al., 2014). Recent studies support a cholinergic anti-inflammatory pathway modulating cell death and inflammation during cerebral ischemia (Pavlov and Tracey, 2005, Vijayaraghavan et al., 2013). The non-selective cholinesterase inhibitor rivastigmine (formerly known as ENA-713), which increases ACh in the synaptic cleft and facilitates cholinergic transmission, reduced ischemia-mediated pyramidal cell loss in the hippocampal CA1 region (Tanaka et al., 1994). Furthermore, elevated total ChE hydrolytic activity has been shown to correlate with the expression of inflammatory markers and cytokines in patients with stroke (Ben et al., 2010), suggesting that hydrolysis of ACh by elevated ChE reduces ACh availability to influence inflammation. We found that the AChE inhibitor Phenserine antagonized glutamate-mediated neuronal death and microglia activation in a mixed neuron/microglia culture; Phenserine-mediated neuroprotection was partially antagonized by methyllycaconitine, indicating its protection was, in part, mediated through the nicotinic α7 receptor. This finding is also supported by a recent study showing that treatment with the nicotinic receptor α7 agonist PNU-282987 reduced expression of activated caspase-3 in stroke brain and reduced neurological deficits (Duris et al., 2011). Phenserine, likewise, significantly reduced brain infarction size in stroke rats (Figure 5). This result cross-validates and extends a prior study demonstrating that pretreatment with Phenserine (1 mg/kg, single dose) reduces stroke volume in rats (Chang et al., 2017). Importantly, Phenserine was administered after ischemic injury in the present study. Mechanisms underpinning neuroprotective actions of Phenserine appear to be cholinergically as well as non-cholinergically mediated.

Posiphen is a stereoisomer of Phenserine with no direct cholinergic (i.e., binding to cholinergic receptors) or indirect cholinergic (i.e., AChE inhibition) activity (Yu et al., 2013). Posiphen has been shown to lower amyloid-beta precursor protein (APP) level in cultured human neuroblastoma cells and in mouse brain (Lahiri et al., 2007). Posiphen, similar to Phenserine, suppresses phytohemagglutinin-induced interleukin-1 mRNA expression in cultured human peripheral blood mononuclear cells (Yu et al., 2013). In a small non-randomized clinical study, Posiphen was found to reduce inflammatory markers (i.e., MCP-1, Complement C3) in the CSF of patients with mild cognitive impairment (Maccecchini et al., 2012). These data suggest that Posiphen has neuroinflammatory modulatory effects in the CNS. We demonstrated here that Posiphen selectively mitigated glutamate-induced neurotoxicity in neuronal cultures. A similar protective response was found after treatment with (+)-N^8^-NorPosiphen. (+)-N^8^-NorPosiphen is a major metabolite of Posiphen found in brain and plasma after systemic administration of Posiphen (Maccecchini et al., 2012, Teich et al., 2018). (+)-N^8^-NorPosiphen, similar to Posiphen, does not bind to cholinergic receptors and is devoid of AChE inhibitory activity (Yu et al., 2013). Notably, the protective effect of Posiphen was not altered by the nicotinic α7 receptor antagonist methyllycaconitine, supporting the premise that Posiphen induces protection through non-cholinergic mechanisms.

Intracellular Ca^++^ homeostasis plays an important role in regulating cell signaling, function, and death. Ischemic/hypoxic injury facilitates glutamate release and increased Ca^++^ levels in the cytoplasm. Blocking NMDA receptors inhibits the inward flow of calcium ions and reduces neuronal injury (Lin et al., 2019). We previously demonstrated that MK801 reduced Glu-activated Ca^++^ influx in neuronal cells expressing the Ca^++^ probe gCaMP5 (Yu et al., 2016). To characterize the direct neuroprotective actions of Posiphen, we evaluated intracellular Ca^++^ homeostasis using the same technique. Posiphen, but not Phenserine, significantly antagonized the NMDA-mediated increase in intracellular Ca^++^. As increasing intracellular Ca^++^ further activates apoptosis, we found that Posiphen treatment reduced TUNEL labeling in cultured neuronal cells and in stroke brain.

An imbalance in cytosolic Ca^++^ can lead to ER stress. The high Ca^++^ in ER is preserved by Ca^++^ regulator proteins (Sacro ER Ca^++^ ATPase protein or SERCA, Ryanodine receptor or RyR, and inositol triphosphate receptor) on the ER membrane (Raffaello et al., 2016). Tg inhibits SERCA and elicits ER stress as a consequence of the change in cytosolic and ER Ca^++^ (Giorgi et al., 2016). Similarly, methamphetamine can induce ER stress (Chen et al., 2019) and increase cytoplasmic Ca^++^ (Yu et al., 2016) in primary neuronal cultures. Co-treatment with the RyR antagonist dantrolene, which regulates ER stress (Li et al., 2005), inhibits the methamphetamine-mediated increase in Ca^++^*i* (Yu et al., 2016). In this study, we found Posiphen not only inhibited Ca^++^*i* but also selectively suppressed Glu and Tg-mediated GLuc-SERCaMP release and Tg-mediated neuronal death in cell culture. Furthermore, Posiphen, but not Phenserine, significantly inhibited Glu or Tg-mediated ER stress in SH-SY5Y cells and primary neurons, as determined by GLuc-SERCaMP release (Henderson et al., 2014). These data hence support the neuroprotective action of Posiphen through inhibition of ER stress in cell culture. Ischemic brain injury also leads to ER stress and can induce further damage *in vivo*. In this study, we overexpressed GLuc-SERCaMP in the cerebral cortex by AAV infection. Gluc immunoreactivity was mainly located in NeuN (+) cells in the cerebral cortex. We demonstrated that ischemia causes release of GLuc in the lesioned cortex, determined by IVIS analysis, suggesting that this ER stress marker was mainly released from neuronal cells. Post-treatment with Posiphen reduced GLuc release from the ischemic brain. The reduction of ER stress by Posiphen was further validated by the down-regulation of the ER stress markers BiP and Chop in the ischemic cortex, suggesting that Posiphen reduces ER stress in ischemic brain. As a consequence of the reduction of ER stress, we found that animals treated with Posiphen had a smaller brain infarction, determined by T2W MRI imaging. In accordance with our *in vitro* cell culture study, Posiphen also reduced TUNEL labeling, morphological activation of microglia, and IBA1 expression in the stroke brain and augmented normal behavioral function in stroke rats.

In conclusion, we demonstrated that Posiphen is neuroprotective against excitatory amino acid and ischemia-mediated brain injury through regulating Ca^++^*i* and ER stress. Early post-treatment with Posiphen reduces neurodegeneration, inflammation, and neurological deficits in stroke animals. Posiphen is currently under clinical trials for Alzheimer's disease (https://clinicaltrials.gov/ct2/show/NCT02925650). It has been reported that people with Alzheimer's disease have a higher risk of stroke (Tolppanen et al., 2013). A high incidence of dementia was also found in the year after a major stroke (Pendlebury and Rothwell, 2019). Posiphen may be a useful therapeutic agent to prevent or treat comorbidity of stroke and dementia, while devoid of cholinergic side effects in patients.

### Limitations of the Study

Ischemic brain injury activates a series of time-dependent pathophysiological responses. Some of these reactions occur shortly after stroke, whereas others can be activated at a much later stage. This study focused on the protective effect of Posiphen at the early phase after injury. We demonstrated that Posiphen reduces injury-mediated Ca^++^i, ER stress, apoptosis in cell culture and in a rat model of stroke. Future studies will need to focus on studying the interaction of Posiphen with delayed degenerative responses and neural repair after ischemic brain injury. Additional experiments will be needed to determine the protective effect of (+)-N8-NorPosiphen, a major metabolite of Posiphen, in stroke brain.

## Methods

All methods can be found in the accompanying Transparent Methods supplemental file.

## Supplemental Information

Document S1. Transparent Methods
