# Supplementary material for: Post-treatment with Posiphen Reduces Endoplasmic Reticulum Stress and Neurodegeneration in Stroke Brain
Source: iScience. 2020 Jan 28;23(2):100866. doi: 10.1016/j.isci.2020.100866 (PMC7013187; doi:10.1016/j.isci.2020.100866)
Supplement: Document S1. Transparent Methods [file mmc1.pdf]

iScience, Volume *23*

Supplemental Information

Post-treatment with Posiphen

Reduces Endoplasmic Reticulum Stress

and Neurodegeneration in Stroke Brain

Seong-Jin Yu, Kuo-Jen Wu, Eunkyung Bae, Yu –Syuan Wang, Chia-Wen Chiang, Li-Wei Kuo, Brandon K. Harvey, Nigel H. Greig, and Yun Wang

Transparent Methods

Animals, AAV vectors, and materials: Adult female timed pregnant and male Sprague-Dawley rats (purchased from the BioLASCO, Taiwan) were used in this study. Experimental procedures followed the guidelines of the "Principles of Laboratory Care" (National Institutes of Health publication No. 86-23, 1996) and were approved by the National Health Research Institutes (Taiwan) Animal Care and Use Committee (Protocol No. 105079-A; 105080). Serotype 1 adeno-associated viral (AAV) vectors were constructed to express GFP (Luo et al., 2013), gCaMP5 (Yu et al., 2016), and GLuc-SERCaMP (Henderson et al., 2015) according to Howard et al with modification (Howard et al., 2008). Vectors were purified by CsCl gradient and titered by quantitative PCR. Viral titers are recorded as viral genome per milliliter. A viral aliquot was thawed, sonicated for 10 s in a 180z ultrasonic cleaner, and diluted to 10 times in PBS with 0.5 mmol/L $MgCl_2$ (Life Technologies). To SH-SY5Y cells overexpressing SERCaMP described in Henderson et al (Henderson et al., 2014), half of their cell media was removed and, thereafter, 5 µL of virus was added to each well for 1hr at 37 °C. After 1hr, fresh media was added in the same volume to replace that previously removed from the cells. Glutamate, NMDA, methyllycaconitine, and Tg were purchased from Sigma-Aldrich (St. Louis, MO, USA). Posiphen, Phenserin, and Posiphen metabolites were synthesized as their water-soluble tartrate salts by the Intramural Research Program, National Institute of Aging, NIH according to Yu et al (Yu et al., 1998, Yu et al., 2013). Chemical characterization confirmed the structure of each, chiral purity and chemical purity (>99.5%).

Primary Cortical Culture: Primary cortical neuron (PCN) cultures (around 65% neurons +35% glia) were prepared from embryonic (E14–15) cortex tissues obtained from fetuses of timed pregnant Sprague-Dawley rats. After removing the blood vessels and meninges, pooled cortices were trypsinized (0.05 %; Invitrogen, Carlsbad, CA, USA) for 20 min at room temperature. After rinsing off trypsin with pre-warmed Dulbecco's modified Eagle's medium (Invitrogen), cells were dissociated by trituration, counted, and plated into 96-well ($5.0x10^4$/well) cell culture plates pre-coated with polyethyleneimine (Sigma-Aldrich). The culture plating medium consisted of neurobasal medium supplemented with 2 % heat-inactivated FBS, 0.5 mmol/L L-glutamine, 0.025 mmol/L L-glutamate and 2 % B27 (Invitrogen). Cultures were maintained at 37 °C in a humidified atmosphere of 5 % CO_2 and

95 % air. The cultures were fed by exchanging 50 % of media with feed media (neurobasal medium) with 0.5 mM L-glutamate, and 2 % B27 with antioxidants supplement on days in vitro (DIVs) 3 and 5. On DIV 7, 10 and 12 cultures were fed with media containing B27 supplement without antioxidants (Invitrogen). Viral transductions were performed on DIV 5 in culture. On DIV 13, cultures were treated glutamate (100 μmol/L), thapsigargin (500 nmol/L), Posiphen (15 μmol/L), Phenerine (15 μmol/L) or vehicle.

Primary rat cortical neuron (PCN) and microglia co-culture: BV2 microglia were cultured separately, detached by 0.05% trypsin-ethylenediaminetetraacetic acid (EDTA, Invitrogen), and centrifuged at 100 g for 5 min. BV2 cells were resuspended in the feeding media containing B27 supplement without antioxidants (-AO, from Invitrogen). The density of surviving cells was counted using a trypan blue assay; cells were plated on the PCN plated-wells at a concentration of $3.0x10^3$/well on DIV 7. The co-cultures were fed with –AO media on DIVs 7 and 10. On DIV 10, cultures were treated glutamate (100 μmol/L) or vehicle. At 48 hr after drug treatment, cells were fixed 4% paraformaldehyde (PFA, Sigma-Aldrich) for 1 hr at room temperature.

Immunocytochemistry: After removing 4% PFA solution, cells were washed with PBS. Fixed cells were treated with blocking solution (2 % BSA, 0.1 % Triton X-100, and 5 % goat serum in PBS) for 1 hr. The cells were incubated for 1 day at 4 °C with a mouse monoclonal antibody against microtubule-associated protein 2 (MAP2; 1:500; Millipore, Billerica, MA) and ionized calcium-binding adaptor molecule 1 IBA1 (1:500; Wako, Richmond, VA, USA) and then rinsed three times in PBS. The bound primary antibody was visualized using AlexaFluor 568 goat anti-mouse secondary (Invitrogen). Images were acquired using a monochrome camera Qi1-mc attached to NIKON TE2000-E inverted microscope by blinded observers.

TUNEL assay for cell culture: Cultures were assayed for DNA fragmentation using a TUNEL-based method (In Situ Cell Death Detection Kit; Roche, Indianapolis, IN). Briefly, 4% PFA fixed cells were permeabilized in 0.1% Triton X-100 in 0.1% sodium citrate for 2 min on ice. To label damaged nuclei, 50 µL of the TUNEL reaction mixture was added to each sample and kept at 37° C in a humidified chamber for 60 min. Procedures for positive and negative controls were carried out as described in the manufacturer’s manual (Roche).

Controls consisted of not adding the label solution (terminal deoxynucleotidyl transferase) to the TUNEL reaction mixture. Images were acquired using a monochrome camera Qil-mc (Diagnostic Instruments, Inc., Sterling Heights, MI, USA) attached to a NIKON TE2000 inverted microscope (Nikon Instruments Inc., Melville, NY, USA). Data were analyzed using NIS-Elements AR 3.2 Software (Nikon Instruments).

SH-SY5Y-GLuc-SERCaMP Cell culture: SH-SY5Y-GLuc-SERCaMP were cultured in a 37°C humidified incubator with 5% CO_2 in DMEM (4.5 g/L D-GLucose) containing 2 mM GlutaMAX, 10% bovine growth serum (Sigma Aldrich), 10 U/mL penicillin (Thermo Fisher Scientific), and 10 µg/mL streptomycin (Thermo Fisher Scientific). Cells were plated at 5 x 10^4 cells per well (100 µL volume). On the next day, media were exchanged into DMEM (4.5 g/L D-GLucose) containing 2 mM GlutaMAX, 1.5% BGS, 10 U/mL penicillin and 10 µg/mL streptomycin before 16-hr drug pre-treatment. Cells were incubated for 48 hr after adding drugs. Media was collected (5 µL) before and after drug treatment, at indicated time points, for enzymatic assay as described above.

Real-time epifluorescence measurement of gCaMP5 in cell culture: Culture plates were placed on a motorized stage (Prior Scientific Inc.) of a Nikon TE2000 inverted microscope. Microscopic images were recorded (1 frame per second) from 10 sec before to 30 sec after drug treatment by an inverted microscope (ECLIPSE Ti2, Nikon, Melville, NY) described in Yu et al (Yu et al., 2016). The intensity of intracellular GFP fluorescence in each well was measured by the NIS Elements AR 5.11 Software.

Gaussia luciferase secretion assay in culture: For luciferase assays (Chen et al., 2019), 5 µl of culture medium was removed and transferred to an opaque-walled plate. GLuc substrate was PBS containing an additional 5 mM NaCl and 10 µM coelenterazine (Sigma-Aldrich). Coelenterazine stock solutions were prepared at 20 mM in acidified methanol (10 µl of 10 N HCl/1 ml of methanol) and stored at -80°C as single-use aliquots. The prepared substrate was incubated at room temperature for 30 min before use. Amount of luciferase was determined using a plate reader with an injector setup (Biotek Synergy HT, Winooski, VT) to immediately read the sample after injection. Typically, 100ul of substrate was injected into the well-containing cell culture medium. For secretion assays, vehicle controls were used in all experiments at concentrations equivalent to the drug treatments.

Animal studies: Adult male Sprague-Dawley rats were anesthetized with chloral hydrate (400 mg/kg). The right middle cerebral artery (MCA) was ligated (MCAo) with a 10-O suture and common carotids (CCAs) were clamped bilaterally by non-traumatic arterial clips to generate focal infarction in the cerebral cortex described in Luo et al (Luo et al., 2009). The suture on the MCA and arterial clips on CCAs were removed after 30-min (for the in vivo ER stress experiment) or 60-min (for infarct volume and behavior experiments) to generate ischemia for reperfusion injury (Luo et al., 2009). Core body temperature was monitored and maintained at 37°C. After recovery from the anesthesia, body temperature was maintained at 37°C using a temperature-controlled incubator. Two drug treatment protocols were used. (1) Posiphen (25 mg/kg/d, i.p.), Phenserine (1 mg/kg/d, i.p) or vehicle was administered to the animals after MCAo for 4 days. We and others have previously reported that the volume of infarction, measured directly by standard triphenyl tetrazolium chloride (TTC) staining, is affected by edema early after the MCAo (Lin et al., 1999, Lin et al., 1993). To reduce the confounding influence of brain edema, brain tissues were collected at 5 days after the MCAo for TTC staining. (2) Posiphen (25 mg/kg/d, i.p.) or vehicle was administered twice, at 1 hour and 1 day after 60-min MCAo. Brain infarction was measured non-invasively by MRI T2Wi. Animals were used for the behavioral assay afterward. Brain tissues were collected to determine the early biochemical changes after MCAo.

Neurological test: Two behavioral tests were used to analyze stroke behavior. (a) Body asymmetry was analyzed using an elevated body asymmetry test (Borlongan et al., 1998, Chang et al., 2002).. Rats were examined for lateral movements/turning when their bodies were suspended 20 cm above the testing table by lifting their tails. The frequency of initial turning of the head or upper body contralateral to the ischemic side was counted in 20 consecutive trials. The maximum impairment in body asymmetry in stroke animals is 20 contralateral turns/20 trials. In non-stroke rats, the average body asymmetry is 10 contralateral turns/20 trials (i.e., the animals turn in each direction with equal frequency). (b) Neurological deficits were evaluated by the Bederson's neurological test (Bederson et al., 1986).

Magnetic resonance imaging: A MRI experiment was performed on a 7T animal scanner (Biospec 70/30 AS, Bruker Biospin MRI, Ettlingen, Germany) with an actively shielded gradient (BGA-12-S, 670 mT/m, 175-ms rise time) described in Wu et al (Wu et al., 2015). A

linear volume resonator was used for RF pulse transmission and an actively decoupled surface coil was used for RF signal reception. The rats were anesthetized with isoflurane (3% induction and 1% maintenance in 30% O_2/ 70% N_2) and secured in a custom-made animal holder with dedicated water heated rat bed, maintaining the rat body temperature. The rate of respiration was monitored by a small animal monitoring unit (SAII Inc., NY, USA). T2-weighted imaging (T2WI) was performed on each rat on day 3 post-stroke. All image slices were acquired in a transverse view of rat brain to cover the whole brain. A fast spin-echo sequence, rapid acquisition with refocused echoes (RARE), was employed and sequence parameters were with a repetition time (TR) of 2742 ms, an echo time (TE) of 33 ms, sa lice thickness of 1 mm, 25 slices, a matrix size of 256 × 256, a number of excitations (NEX) of 4, and a field of view (FOV) of 25 mm × 25 mm. The acquisition time was approximately 6 min. For brain infarction measurement, the region-of-interests (ROIs) identified in hyper-intensity regions in T2WI were manually determined by two of the authors (CWC and LWK). The area of infarction was calculated by averaging ROIs in every two adjacent image slices of the brain from bregma +10 mm to bregma -14 mm. Lesion volume (LV) was calculated by the summation of total area infarction.

***In vivo* delivery of AAV- GLuc-SERCaMP and imaging of GLucSERCaMP using the In Vivo Imaging System (IVIS)**: Adult rats were anesthetized and were placed in a stereotaxic frame (Stoelting). AAV-gLuc-ASRTDL [2 µL of $5x10^9$ viral genomes/µL per site) was delivered into 3 cortical sites in the distribution of the MCA. The stereotaxic coordinates were: AP 1.2 mm, ML 5 mm, DV -3.5 mm (site1); AP -0.3 mm, ML 5 mm, DV -3.5 mm (site 2); AP -1.8 mm, ML 5.5 mm, DV -3.5 mm (site 3) according to Airvaara et al (Airavaara et al., 2010). The rate of infusion (1 µL/min) was adjusted by a microprocessor controlled injector mounted to the stereotaxic frame (UMP4; World Precision Instruments, Sarasota, FL, USA). The needle remained in the brain for 2 min after the injection and then was slowly removed. After recovery from anesthesia, animals were housed in their home cages. At 2 weeks after viral infection, animals received a 30-min MCAo. One day after the MCAo, animals were anesthetized with isoflurane and then transferred to an IVS Lumina 2 Imaging System chamber (Caliper Life Sciences, Cheshire, United Kingdom). Coelenterazine (Regis Technologies, Morton Grove, IL) was given to the animals intraveneoulsy through the tail vein at a dose of 100 µg/150 µL/rat. GLuc fluorescence images in brains were acquired

using a CCD camera. The intensity of photons collected through IVIS was translated to false color images with strong fluorescence in yellow and was quantified by the imaging software.

TTC staining of infarction volume. Brains were removed, sliced into 2.0-mm thick sections, included in 2% wt/vol TTC, fixed in 4% wt/vol PFA, and then digitally scanned. The area of infarction was analyzed by observers blinded to treatment group (Shen et al., 2005, Tomac et al., 2002).

Immunohistochemistry: Animals were anesthetized and perfused transcardially with saline followed by 4% PFA in phosphate buffer (PB; 0.1 mol/L; pH 7.2), they were post-fixed for 18–20 hr and then transferred to 20% sucrose in 0.1 M PB for at least 16 hr. Serial sections of brains were cut at 30-μm thickness on a cryostat (model: CM 3050 S; Leica, Heidelberg, Germany). Brain sections were rinsed in PB and were blocked with 4% bovine serum albumin (Sigma-Aldrich) with 0.3% Triton X-100 (Sigma-Aldrich) in 0.1 mol/L PB. Brain slices were then incubated with primary antibodies against Gaussia luciferase (1:100; Cat: E8023S; New England Biolabs, MA, USA), GFAP (monoclonal 1:100; Cat: MAB360; Merck, NJ, USA), NeuN (monoclonal 1:100; Cat: MAB377; Merck, NJ, USA), or IBA1 (monoclonal 1:100, Chemicon, Billerica, MA, USA) at 4°C overnight. Sections were rinsed in 0.1 mol/L PB and incubated in Alexa Fluor 488 secondary antibody solution (1:500; Molecular Probes, Eugene, OR, USA). Control sections were incubated without the primary antibody. Brain sections were mounted on slides and coverslipped. Confocal analysis was performed using a Nikon D-ECLIPSE 80i microscope (Nikon Instruments, Inc., Tokyo, Japan) and EZ-C1 3.90 software (Nikon, Tokyo, Japan). The optical density of IBA1 immunoreactivity was quantified in three consecutive brain sections with a visualized anterior commissure in each animal. Six photomicrographs were taken along the perilesioned region per brain slices; IBA1 optical density was analyzed by NIS Elements AR 3.2 Software (Nikon) and was averaged in each brain for statistical analysis. All immunohistochemical measurements were performed by blinded observers.

TUNEL labeling: Apoptotic cell death was detected by terminal deoxynucleotidyl transferase (TdT), which catalyzes polymerization of labeled nucleotides to free 3’-OH DNA ends in a template-independent manner (TUNEL reaction), according to the manufacturer's protocols

(In situ cell death detection kit, Cat. No. 11684795910, Roche). Brain sections were attached to a glass slide and were air dried at room temperature for 20 min. Sections were then washed with 0.1M PB for 30 min and incubated in permeabilization solution for 2 min on ice. 50 µl of TUNEL reaction solution was added to the samples, and incubated samples were placed in a humidified atmosphere for 60 min at 37°C in the dark. Sections were rinsed in 0.1M PB and were cover-slipped. For a negative control, sections were incubated with the same volume of label solution instead of the TUNEL reaction solution. Confocal analysis was performed using a Nikon D-ECLIPSE 80i microscope (Melville, NY, USA) and EZ-C1 3.90 software. The optical density of TUNEL positive cell was quantified in three consecutive brain sections with a visualized anterior commissure in each animal. Six photomicrographs were taken along the perilesioned region per brain slices; TUNEL positive optical density was analyzed by NIS Elements AR 3.2 Software (Nikon) and was averaged in each brain for statistical analysis.

Western analysis: Brain tissue was homogenized in RIPA lysis buffer (Cell Signal). The homogenate was centrifuged at 13200 rpm for 10 min at 4 °C, and the supernatant was collected. A bicinchoninic acid (BCA) protein assay was performed using bovine serum albumin to determine protein concentrations. The samples were diluted with RIPA buffer according to the BCA protein assay. Gels were transferred to a PVDF membrane after electrophoresis. The membranes were blocked in Odyssey blocking buffer (LI-COR) for 2 h or 5% milk (ECL) for 1 h at room temp. The blots were then probed with primary antibodies against ionized calcium-binding adapter molecule 1 (IBA1, 1: 1000, Wako) or immunoglobulin heavy chain binding protein (BiP, 1:1000, Cell signaling) at 4 °C for overnight, and actin (1:5000, Novus) at room temp for 1 h. The membrane was then incubated with an IRDye® 800CW Goat anti-Rabbit (1:2500 for IBA1, LI-COR), IRDye® 680LT Goat anti-Mouse (1:5000 for actin, LI-COR) or horseradish peroxidase (HRP)-conjugated secondary antibody (Jackson lab) at room temp for 1 h, followed by washing with 0.1% Tween-20 (in TBS) five times for 5 min each. IBA1 and actin immunoreactivities were scanned by an infrared imaging system (Odyssey, LI-COR) and were quantified using Image

Studio Lite Ver 5.2 (LI-COR). The light emission signal of the BiP and actin was generated by using a Western Lightning Plus-ECL (PerkinElmer) and then displayed on X-ray film (Cat. No. NEF596, Kodak). ECL-Band intensity was quantified using Image J. Finally, the amount of IBA1 or BiP was normalized with actin on the same membrane.

Quantitative Reverse Transcription –PCR (qRTPCR): Brain cortical tissues were collected for qRT-PCR analysis at 2 days after the MCAo (Yu et al., 2017). An additional 8 non-stroke rats were used as the control group. Total RNAs were isolated by use of a RNeasy Mini Kit (Qiagen, #74106) and cDNAs were synthesized from 1ug total RNA by use of RevertAid H Minus First Strand cDNA Synthesis Kit (Thermo Scientific, #K1631). The TaqMan Gene Expression Assays primer for specifically detecting Rat Beta-actin (#Rn00667869_m1) and GAPDH (#Rn01775763_g1) were purchased from Thermo Scientific. Quantitative Real-Time PCR (qRT-PCR) was carried out using TaqMan Fast Advanced Master Mix (Life Technologies, #4444557) and Applied Biosystems 7500 Fast Real-Time PCR System. The expressions of PERK, CHOP, and BiP mRNA were measured by using SYBR (Luminaris Color HiGreen Low ROX qPCR Master Mix; ThermoScientific). The primers used were as follows:
PERK (GenBank Acc.), forward: 5’- GAAGTGGCAAGAGGAGATGG -3’ and reverse: 5’- GAGTGGCCAGTCTGTGCTTT -3’; CHOP (GenBank Acc.), forward: 5’- ACCACCACACCTGAAAGCAG -3’, reverse: 5’- AGCTGGACACTGTCTCAAAG -3’; BiP (GenBank Acc.), forward: 5’- TCGACTTGGGGACCACCTAT -3’ and reverse: 5’- GCCCTGATCGTTGGCTATGA -3’. Expression and normalization of the target genes, PERK, CHOP, and BiP were calculated relative to the endogenous reference gene (Beta-actin and GAPDH averages) with a modified delta-delta-Ct algorithm that takes gene-specific amplification efficiency into account for accurate calculation. All experiments were carried out in duplicate.

Statistics: Data are presented as mean ± s.e.m. Unpaired t-test, 1 or 2-way ANOVA, 1 or 2-way ANOVA on rank were used for statistical comparisons, with a significance level of $p<0.05$. In the event of multiple comparisons, posthoc Newman-Keuls test (NK test) was performed.

Reference

Airavaara, M., Chiocco, M.J., Howard, D.B., Zuchowski, K.L., Peranen, J., Liu, C., Fang, S., Hoffer, B.J., Wang, Y., and Harvey, B.K. (2010). Widespread cortical expression of MANF by AAV serotype 7: localization and protection against ischemic brain injury. Exp. Neurol. *225*, 104-113.

Bederson, J.B., Pitts, L.H., Tsuji, M., Nishimura, M.C., Davis, R.L., and Bartkowski, H. (1986). Rat middle cerebral artery occlusion: evaluation of the model and development of a neurologic examination. Stroke *17*, 472-476.

Borlongan, C.V., Hida, H., and Nishino, H. (1998). Early assessment of motor dysfunctions aids in successful occlusion of the middle cerebral artery. Neuroreport *9*, 3615-3621.

Chang, C.F., Morales, M., Chou, J., Chen, H.L., Hoffer, B.J., and Wang, Y. (2002). Bone morphogenetic proteins are involved in fetal kidney tissue transplantation -induced neuroprotection in stroke rats. Neuropharmacology *43*, 418-426.

Chen, Y.H., Bae, E., Chen, H., Yu, S.J., Harvey, B.K., Greig, N.H., and Wang, Y. (2019). Pifithrin-alpha reduces methamphetamine neurotoxicity in cultured dopaminergic neurons. Neurotox Res *36*, 347-356.

Henderson, M.J., Wires, E.S., Trychta, K.A., Richie, C.T., and Harvey, B.K. (2014). SERCaMP: a carboxy-terminal protein modification that enables monitoring of ER calcium homeostasis. Mol Biol Cell *25*, 2828-2839.

Henderson, M.J., Wires, E.S., Trychta, K.A., Yan, X., and Harvey, B.K. (2015). Monitoring Endoplasmic Reticulum Calcium Homeostasis Using a Gaussia Luciferase SERCaMP. J Vis. Exp. *103*, 53199.

Howard, D.B., Powers, K., Wang, Y., and Harvey, B.K. (2008). Tropism and toxicity of adeno-associated viral vector serotypes 1,2,5,6,7,8,9 in rat neurons and glia *in vitro*. Virology *372*, 24-34.

Lin, S.Z., Hoffer, B.J., Kaplan, P., and Wang, Y. (1999). Osteogenic protein-1 protects against cerebral infarction induced by MCA-ligation in adult rats. Stroke *30*, 126-133.

Lin, T.N., He, Y.Y., Wu, G., Khan, M., and Hsu, C.Y. (1993). Effect of brain edema on infarct volume in a focal cerebral ischemia model in rats. Stroke *24*, 117-121.

Luo, Y., Kuo, C.C., Shen, H., Chou, J., Greig, N.H., Hoffer, B.J., and Wang, Y. (2009). Delayed treatment with a p53 inhibitor enhances recovery in stroke brain. Ann. Neurol. *65*, 520-530.

Luo, Y., Shen, H., Liu, H.S., Yu, S.J., Reiner, D.J., Harvey, B.K., Hoffer, B.J., Yang, Y., and Wang, Y. (2013). CART peptide induces neuroregeneration in stroke rats. J. Cereb. Blood Flow Metab *33*, 300-310.

Shen, H., Chen, G.J., Harvey, B.K., Bickford P.L., and Wang, Y. (2005). Inosine reduces ischemic brain injury in rats. Stroke *36*, 654-659.

Tomac, A.C., Agulnick, A.D., Haughey, N., Chang, C.F., Zhang, Y., Bäckman, C., Morales, M., Mattson, M.P., Wang, Y., Westphal, H. et al (2002). Effects of cerebral ischemia in mice deficient in Persephin. Proc. Natl. Acad. Sci. U. S. A *99*, 9521-9526.

Wu, K.J., Yu, S.J., Chiang, C.W., Cho, K.H., Lee, Y.W., Yen, B.L., Kuo, L.W., and Wang, Y. (2015). Transplantation of human placenta-derived multipotent stem cells reduces ischemic brain injury in adult rats. Cell Transplant. *24*, 459-470.

Yu, Q., Greig, N.H., Holloway, H.W., and Brossi, A. (1998). Syntheses and anticholinesterase activities of (3aS)-N1, N8-bisnorphenserine, (3aS)-N1,N8-bisnorphysostigmine, their antipodal isomers, and other potential metabolites of phenserine. J. Med. Chem. *41*, 2371-2379.

Yu, Q.S., Reale, M., Kamal, M.A., Holloway, H.W., Luo, W., Sambamurti, K., Ray, B., Lahiri, D.K., Rogers, J.T., and Greig, N.H. (2013). Synthesis of the Alzheimer drug Posiphen into its primary metabolic products (+)-N1-norPosiphen, (+)-N8-norPosiphen and (+)-N1, N8-bisnorPosiphen, their inhibition of amyloid precursor protein, alpha-Synuclein synthesis, interleukin-1beta release, and cholinergic action. Antiinflamm. Antiallergy. Agents Med. Chem. *12*, 117-128.

Yu, S.J., Airavaara, M., Wu, K.J., Harvey, B.K., Liu, H.S., Yang, Y., Zacharek, A., Chen, J., and Wang, Y. (2017). 9-cis retinoic acid induces neurorepair in stroke brain. Sci Rep *7*, 4512.

Yu, S.J., Wu, K.J., Bae, E.K., Hsu, M.J., Richie, C.T., Harvey, B.K., and Wang, Y. (2016). Methamphetamine induces a rapid increase of intracellular Ca^{++} levels in neurons overexpressing GCaMP5. Addict Biol *21*, 255-266.
