# Supplementary material for: Post-treatment with Posiphen Reduces Endoplasmic Reticulum Stress and Neurodegeneration in Stroke Brain
Source: iScience. 2020 Jan 28;23(2):100866. doi: 10.1016/j.isci.2020.100866 (PMC7013187; doi:10.1016/j.isci.2020.100866)
Supplement: Document S2. Figure S1 [file mmc2.pdf]

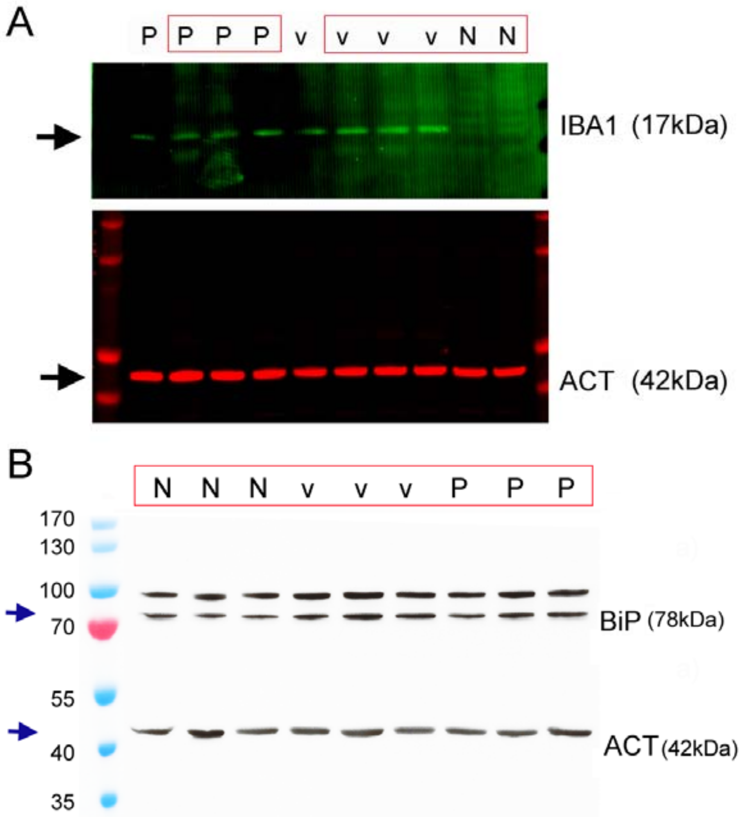

The full-length Western blots of gels of IBA1, BiP and β-actin presented in Figs 7B1 and 8E of the main article. Blots were probed with primary antibodies against ionized calcium-binding adapter molecule 1 (IBA1, 1: 1000, Wako) or BiP (1:1000, Cell signaling) at 4°C for overnight, or actin (1:5000, Novus) at room temp for 1 h. The membrane was then incubated with an IRDye® 800CW Goat anti-Rabbit (1:2500 for IBA1, LI-COR), IRDye® 680LT Goat anti-Mouse (1:5000 for actin, LI-COR) or horseradish peroxidase (HRP)-conjugated secondary antibody (Jackson lab) at room temp for 1 h. (A) IBA1 (top panel) and β-actin (lower panel) immunoreactivities were scanned by an infrared imaging system (Odyssey, LI-COR). (B) The light emission signal of the BiP and actin was generated by using a Western Lightning Plus-ECL (PerkinElmer) and then displayed on X-ray film. Red rectangles represent lanes selected for Figs 7B and 8E. N: non-stroke, P: Posiphen +stroke, v:vehicle+stroke
